# Supplementary figures and images for: Predicting regional influenza epidemics with uncertainty estimation using commuting data in Japan
Source: PLoS One. 2021 Apr 22;16(4):e0250417. doi: 10.1371/journal.pone.0250417 (PMC8062106; doi:10.1371/journal.pone.0250417)

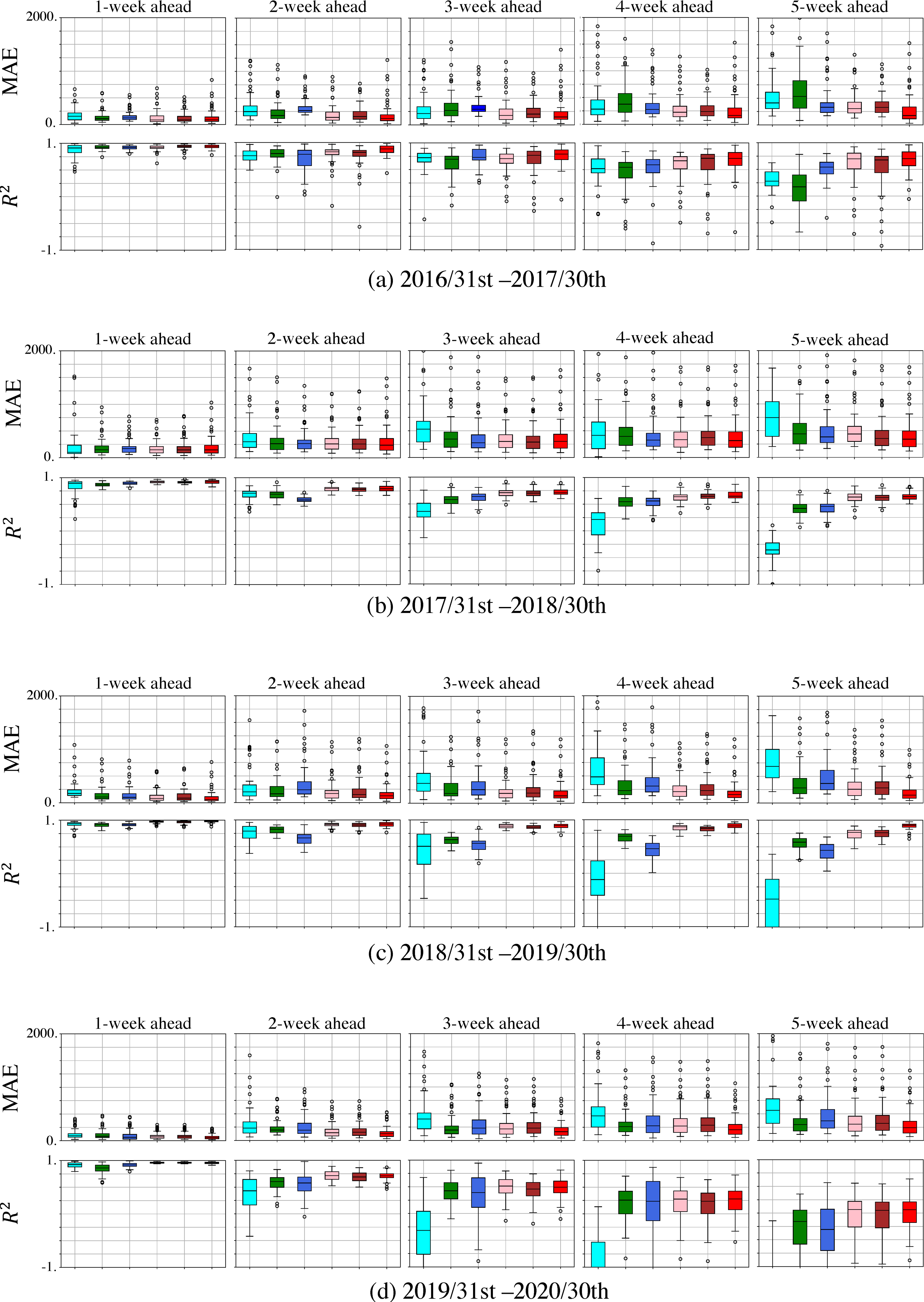

Supplement: S1 Fig — This figure shows the boxplots of the distribution of the prediction scores (MAE and R2) in each prefecture for the compared models. Each colored box indicates a different model; from left to right: VAR (cyan), LSTM (green), CNNRNN-Res (blue), GCN+S2s w/ AD (pink), GCN+S2s w/ DD (brown), and GCN+S2s w/ PF (red). The black center line in each box indicates the median value; the top and bottom of each box indicate the upper and lower quartiles, respectively; the whiskers indicate the maximum and minimum values; and the other points indicate outliers. For visualization, only MAE scores from 0 to 2000 and R2 scores from -1.0 to 1.0 are shown. (TIF) [file pone.0250417.s001.tif]
