# Supplementary material for: Predicting regional influenza epidemics with uncertainty estimation using commuting data in Japan
Source: PLoS One. 2021 Apr 22;16(4):e0250417. doi: 10.1371/journal.pone.0250417 (PMC8062106; doi:10.1371/journal.pone.0250417)
Supplement: Attachment — Submitted filename: Review for PONE-D-20-20268.pdf [file pone.0250417.s002.pdf]

Review for PONE-D-20-20268

Title: Predicting Regional Influenza Epidemics with Uncertainty Estimation using Commuting Data in Japan.

Comment

The study proposed a graph convolutional network (GCN) based prediction model to predict influenza epidemic, which temporal trend has a 'periodicity' pattern. The model incorporate commuting data (from 2015 census) and spatial adjacency relationship as the interaction between (47) areas in the model, and used 3 flu seasons (from 2016 to 2019) as the study periods, to test and compare their model with other methods. With the three research questions analyses, they concluded that their proposed model outperform the other previous models. While the idea, method and analyses are interesting, the current status of the manuscript is yet to reach publishable quality. Therefore, I would recommend major revision. My concerns were listed as follow.

Major concerns:

1. One key contribution of the study should be the consideration of 'periodicity in a time series' (page 2, line 51) in the model, which was neglected in the previous Zhu et al. [18] Encoder-Decoder model. But, the authors did not explain what is it, and why it is important. Since the study did not use time-dependent dynamic commuting data, I would 'guess' the periodicity is in the weekly disease data. The authors should not let readers to guess, thus they should explain and clarify the 'periodicity' term where they first mention it, and emphasize the consequence of neglecting it; and which would help emphasizing the contribution of this study.
2. According to the dataset description, the commuting data is in 'the daily average number of commuters from one area to another area'. Is this dataset differentiate weekdays/weekends, or from Monday to Sunday? The time unit for the model is by weekly basis, how did the daily data converted to weekly before the 'min-max normalization'?
3. Also about the description of the commuting data (page 9, line 276-282), the authors describe the number of commuters as 'inflow of commuting data', e.g. the 270,000 and 135,000, as the number of commuters from one area to another. Based on the terminology from graph theory and social network analysis, the term 'inflow' could indicate the total number of people/commuters go to a target area, e.g. the total number of people go into Tokyo from any area; and the counterpart 'out-flow' could mean the total people leaving from the area. The usage of term 'inflow' is misleading.
4. Following the #3 point, the input data for the model should be a weighted directed matrix (as suggested in figure 1). Commuting data is expected to be the number of people commute from the home area to work area. The people eventually will go back to their home in daily basis, i.e. a reversed direction flow relationships, or transpose matrix of flow matrix. Why the reverse direction of commuting flow is not considered and processed in the model? And, why direction of flow matters in the machine-learning based model?
5. Both figures 5 and 6 suggested that all models' predictions were lower than the true values at the peak of trends, especially the second peak (near 2018 10th). Why they all failed to capture the peak values? Why LSTM's peaks were almost all earlier than the true value, whereas CNN-Res were always later?
6. In page 3 line 91, the authors claimed that 'Our study is the first to predict the influenza volume in detail on a large area...'. But in fact, the model considered only 47 areas, which is not a large number and is a low resolution for the whole country. Practically speaking, the 47 areas (assumably prefectures) might be enough for national level management, but they are too large for local disease control or health management, therefore not so useful for 'regional public health organizations' (page 15 line 459). Is this model applicable to smaller areas (higher resolution, e.g. municipal)? If so, what should be prepared and which part should be modified; if not, why?
7. Following previous point, is it possible to extend/apply the model to be used in early warning system?
8. From the view of spatial epidemiology, the disease spread from one place to another, through droplets or direct/indirect physical interactions (etc.) and through the flow of the infected people. The infectious process is described as SIR model, which has (at least) three conditions: susceptible, infected, and recovered. The infected person go through the SIR process, and thus a time-lag is expected in the process, i.e. from susceptible to infected, and from infected to recovered. How does this machine-learning based model(s) handle the complicated SIR (or SEIR, SLIR, SIS, etc.) process and the time-lag effect?

Suggestions and minor concerns:

1. Table 2 presented the average MAE and R-squared of 47 areas. While the average values shows that their model (GCN+S2s w/ PF) are in overall outperform other models, the average values may be misleading by outliers. Thus, showing the distribution of the 47 values were needed, e.g. with std or boxplots. I believe these results could be presented using a set of boxplots (3 MAE and 3 R-squared), with vertical axis showing the MAE or R-squared, horizontal-axis showing the 1-to-5-weeks, and six boxes (different colors) for each week showing the values for 47 areas for the six models. Line plot with error bars can also be used to show the average and plus-minus standard deviation if boxplot is not clear.
2. Following previous point, it should be possible to calculate the MAE and R-squared in aggregated (national) level, instead of average of 47, and the national level results shall also be useful for discussion.
3. The comparative model (GCN+S2s w/ AD) considered only the adjacent relations between areas (polygon shapes of the 47 areas). In transportation and spatial analysis, the strength of interaction between cities (e.g. flows) can be estimated mainly using gravity model or radiation model. In simple words, the interaction strengths were higher between closer cities, and lower between farther cities, i.e. distance decay effect. What if adding another comparative model that calculate the inversed distance as the weight matrix?
4. Page 12 line 378, what is 'examples of learned'. What do the colors means in Figure 3. Tokyo and Aichi were in the 'min' values, which should means that the interactions from Nara to Tokyo/Aichi are low, thus not important and could be ignored?
5. Figure 4, consider adding legends on maps. And since the authors presented a map for a year, it would be better to show the 'improvement' percentage in all 47 areas with color ramp, and maybe used colored thick borders to highlight the highest and lowest five areas.
6. Figures 3 and 4, what is the purpose of the small squares at the corner of each map (not the Hokkaido area)?
7. Table 1, consider align the second column (Definitions or Descriptions) to left.
8. Figures 5 and 6 consider changed x-label to Weeks from Date.
9. Finally, while the content is quite rich, the English writing in the manuscript is not publishable; some of the sentences needs to read twice or more to understand/guess the authors meaning, e.g. the above point 4 (examples of learned?), page 13 line 416 (beginning of epidemics?). It would be difficult for readers to understand the idea/method/uniqueness/contribution of the study, and possibly lead to misunderstanding. Please revise the writing.
