# Supplementary material for: Predicting regional influenza epidemics with uncertainty estimation using commuting data in Japan
Source: PLoS One. 2021 Apr 22;16(4):e0250417. doi: 10.1371/journal.pone.0250417 (PMC8062106; doi:10.1371/journal.pone.0250417)
Supplement: Attachment — Submitted filename: Plos_one_reply.pdf [file pone.0250417.s003.pdf]

Dear Dr Tzai-Hung Wen
Academic Editor, PLOS ONE

Thank you for inviting us to submit a revised draft of our manuscript (PONE-D-20-20268) entitled “Predicting Regional Influenza Epidemics with Uncertainty Estimation using Commuting Data in Japan” to the PLOS ONE. We also appreciate the time and effort you and each reviewer has dedicated to providing insightful feedback on ways to strengthen our paper. Thus, it is with great pleasure that we re-submit our manuscript for further consideration. We have incorporated changes that reflect the detailed suggestions you have graciously provided. We also hope that the edits and responses we have provided satisfactorily address all the issues and concerns you and the reviewers have noted. In this rebuttal letter, the comments or suggestions from you and the reviewers are inside the black frames, our responses follow them, and our revisions in the manuscript are shown in italics.

Best regards,
Taichi Murayama

To the editor:

1. Please ensure that your manuscript meets PLOS ONE's style requirements, including those for file naming.

Thank you for your comment. We have ensured that our manuscript meets your style requirements. Please let us know if anything needs to be revised.

2. Please provide more information on the data used in your study. Specifically, please report the following in your methods section.

Thank you for your comment. We revised the dataset description in the "Datasets" subsection of the "Experiment" section to provide a detailed explanation of the data as follows:
We added the time range of data, last access date, and URL to the description of the influenza data. In addition, we added the last access date and URL to the description of the commuting data.

P10 Lines 267-295
Datasets
Influenza data
We used data based on the weekly number of patients with influenza symptoms for each prefecture in Japan reported by the National Institute of Infectious Diseases (NIID). NIID reports aggregated information related to influenza in its weekly reports[55] to warn about infection outbreaks. These reports are delayed by approximately seven days from the date of the original clinical reports by physicians (because of the time necessary to aggregate clinical information from different health authorities in each prefecture). We mixed all subtypes of influenza data, and accumulated the number of influenza patients from the 37th week of 2012 to the 30th week of 2020.

Commuting data
Spatiotemporal models typically use adjacency and distance between observation points to model geographic information. However, human mobility is strongly linked with the transmission of infectious diseases (such as droplets and contact infections). Therefore, this study uses commuting data, instead of adjacency data, as geographic information (Figure 1). Adding commuting data to our model can better capture epidemic situation in a region, which is important for public health organizations.

To consider inter-regional flows of people, we used commuting data from 47 prefectures in Japan. The data were provided by the national census report [56] in 2015. The provided data include the single daily average numbers of commuters from one prefecture to another, throughout all the days of the weeks. For the experiment in each year, we represent the data as a graph G = (V, E, W) in the proposed model because the national census report provides only the number of commuters, regardless of the year. We divided the number of commuters by the maximum number of commuters between every prefecture pair, which is known as the min-max normalization to graph-edge information, such as W. The maximum number of commuters (270,000) travels from Kanagawa prefecture to Tokyo prefecture. Moreover, 135,000 commuters travel from Osaka to Nara. The edge weight, representing commuters

traveling from Osaka to Nara, is shown as 0.5 (=135,000/270,000) in the graph, after applying min-max normalization.

[55] The National Institute of Infectious Diseases [cited 21 Oct 2020]. Available from: https://www.niid.go.jp/niid/ja/idwr.html.
[56] The National Census Report of 27th year of the Heisei period [cited 21 Oct 2020] Available from: https://www.stat.go.jp/data/kokusei/2015/kekka.html.

3.1 Please provide an amended Funding Statement declaring this commercial affiliation, as well as a statement regarding the Role of Funders in your study. If the funding organization did not play a role in the study design, data collection and analysis, decision to publish, or preparation of the manuscript and only provided financial suppor in the form of authors' salaries and/or research materials, please review your statements relating to the author contributions, and ensure you have specifically and accurately indicated the role(s) that these authors had in your study.

Thank you for your comment. The funding organization only provides financial support in the form of authors' salaries or research materials. We agree with the following statement: "Could you please change the online submission form as follows?"

"The funder provided support in the form of salaries for Dr. Nobuyuki Shimizu and Mr. Sumio Fujita, but did not have any additional role in the study design, data collection and analysis, decision to publish, or preparation of the manuscript. The specific roles of these authors are stated in the 'author contributions' section."

3.2. Please also provide an updated Competing Interests Statement declaring this commercial affiliation along with any other relevant declarations relating to employment, consultancy, patents, products in development, or marketed products, etc.

Thank you for your comment. We confirmed that our adherence to PLOS ONE policies does not change, and sharing of data and/or materials has no restrictions. We added the following sentence: "The authors have declared that funding from a commercial source, Yahoo Japan Corporation, does not alter our adherence to PLOS ONE policies on sharing data and materials." in the Competing Interests Statement. Could you please change our Competing Interests Statement as above?

We will add an updated Funding Statement and Competing Interests Statement in our cover letter.

4. We note that Figure 1, 2, 3, 4 in your submission contain map images which may be copyrighted. All PLOS content is published under the Creative Commons Attribution License (CC BY 4.0), which means that the manuscript, images, and Supporting Information files will be freely available online, and any third party is permitted to access, download, copy, distribute, and use these materials in any way, even

commercially, with proper attribution. For these reasons, we cannot publish previously copyrighted maps or satellite images created using proprietary data, such as Google software (Google Maps, Street View, and Earth). For more information, see our copyright guidelines:

Thank you for your consideration of our images. We replaced all the images related to the map of Japan in our figures (Figure 1 - 4) by images under Creative Commons, which are supplied by CartoDB Positron. (https://carto.com/basemaps/).

To Reviewer #1:

1. The reference number order in the article is messy, please renumber in numerical order.

Thank you for your comment. In accordance with the comment, we have arranged the references in numerical order.

2. In lines 265 to 269, the author made the following explanation for Influenza data: "We use data for the weekly number of patients with influenza symptoms...of clinical information". However, Influenza's disease transmission and virus patterns are very complicated, and there may be asymptomatic infections, which are important parameters that cannot be captured in the data. The following points should be further explained:

2-a. Which subtype of Influenza is the simulation study for?

Thank you for your question. Our research does not distinguish any specific influenza subtypes; however, it handles patients with all subtypes together.
We added such an explanation in the "Datasets" subsection of the "Experiment" section as follows:

P10 Lines 267-276
Datasets
Influenza data
We used data based on the weekly number of patients with influenza symptoms for each prefecture in Japan reported by the National Institute of Infectious Diseases (NIID). NIID reports aggregated information related to influenza in its weekly reports[55] to warn about infection outbreaks. These reports are delayed by approximately seven days from the date of the original clinical reports by physicians (because of the time necessary to aggregate clinical information from different health authorities in each prefecture). We mixed all subtypes of influenza data, and accumulated the number of influenza patients from the 37th week of 2012 to the 30th week of 2020.

2-b. How to set the parameters that meet this Influenza subtype, especially the E (Exposed) infected persons in the SEIR model in the compartmental models?

Thank you for your comment. As our model is based on a machine learning approach, it significantly differs from compartmental models. Examples of compartmental models are SIR and SEIR, which consider **susceptible**, **exposed**, **infectious**, and **recovered** as the states of patients to understand disease dynamics.
In contrast, our model based on machine learning focuses only on observed **infectious** cases, i.e., reported numbers of patients from health authorities once a week. In particular, it focused

on the accurate prediction of future numbers of observed **infectious** cases by leveraging the past numbers of observed **infectious** cases and other resources, such as commuting data. Therefore, our model does not expressly consider other compartments, i.e., **susceptible**, **exposed**, and **recovered**, and does not need to set the parameters for **exposed** cases or other compartments.

Our model aims to achieve the highest prediction accuracy of observed **infectious** cases in the future among other models, including compartmental models. We added an explanation on the difference between machine learning methods (such as ours) and compartmental models (such as SEIR) in the "Influenza prediction" subsection of the "Related work" section as follows:

P3 Lines 67-81

Related work

Influenza prediction

Influenza prediction methods can be broadly classified into three categories: compartmental model-based, statistical and time series, and machine-learning.

Compartmental models include ``susceptible--infected--removed'' (SIR) [10] and incidence decay with exponential adjustment [11] models. They differ from statistical and machine-learning methods because they set suitable parameters for each compartment and focus on understanding disease dynamics. Statistical and time-series methods include the autoregression-integrated moving average [12] and generalized autoregressive moving average [13] methods. In particular, the autoregression with Google search method (ARGO method) [14], which is based on linear regression using the input data of the Google search time series and historical influenza-like illness data, has exhibited superior results for flu forecasting [15-17]. Our GCN-based model is based on machine learning. Other examples of machine-learning methods include linear regression [18,19], random forest [20], Gaussian process [21], and long short-term memory (LSTM) [7,8,22].

2-c. Generally, people infected with Influenza are very likely to be infected again after recovery. How to correctly evaluate the number of people infected after recovery?

As mentioned above, our model, being a machine learning method, differs from compartmental models, which focus on understanding influenza dynamics. Nevertheless, our model aims to achieve higher predictive performance of the number of patients in the future, and it considers only observed **infectious** cases.

In other words, it considers the number of people infected after recovery as a part of the total reported number of infectious cases without setting parameters. Next, it predicts the future number of all patients, irrespective of their states. To specifically estimate the number of people infected after recovery is out of the scope of our current study.

2-d. Since this study is based on Influenza, it does not seem to be based on a certain subtype. However, when all Influenza subtypes have been mixed and discussed, how to verify the experimental results with actual data? (Especially the data of E infected persons cannot be obtained)

Our model predicts the future number of flu patients, as reported in the "Experiments" section, and we discuss the experimental results based on actual data. Regarding influenza subtypes, our model might predict the future number of patients of a certain subtype if trained for a specific subtype. However, as our model does not consider each state (such as **exposed**), except observed **infectious** cases, for the same reason as explained in (2-b), it does not make much sense to distinguish subtypes with respect to the prediction of the total number of patients of all subtypes.
We are convinced that the parameter setting for each subtype is important for understanding influenza dynamics. Nonetheless, given the purpose of our current study, which aims to predict the total number of influenza patients in the future, it is beyond the scope of our current study.

3. The author uses the commuting network (Fig.1) to simulate the spread of disease in this study. However, generally speaking, the commuting network may use different means of transportation such as highways, trains, high-speed rails, planes, and ships. Sometimes the disease may spread on a large scale because an infected person takes a plane or a ship, and the plane and ship play a long-distance transmission role. However, the communicating network of Fig.1 seems to be too simple, and there is no network layer for airplanes and ships, but the simulation result (Fig.5) is very close to the true value.

Thank you for your comment. Commuting data, as one of our resources, was collected from the national census report of Japan. The provided data include the daily average number of commuters from one area to another by whichever means they commute. Therefore, if a person uses planes or ships for commuting purposes, such movements are counted in our commuting data.
You precisely indicated that the data do not include unusual movements, such as travel or business trips, although the prediction results (Fig. 5 and Table 2) are very close to the actual values. This is because unusual movements are less common than commuting. Hence, unusual movements are not good signals for predicting the total number of patients. It is also very likely that possible influenza patients refrain from moving long distances due to their poor health conditions. Although returning travelers from distant infected regions might bring back influenza viruses, such events are sparse and accidental; thus, they would not constitute a large body of signals for prediction purposes. However, our machine learning method is in a good position to achieve sufficient prediction even with incomplete information about people's flow. We think this is a good feature of machine learning methods because incomplete and noisy information is common in practical situations.

3. Can the author further explain the information acquisition and use of the commuting network in this study?

Thank you for your comment. We have added a more detailed description of information acquisition and the use of the commuting network in the "Datasets" subsection of the "Experiment" section as follows:

P10 Lines 277-295

Commuting data

Spatiotemporal models typically use adjacency and distance between observation points to model geographic information. However, human mobility is strongly linked with the transmission of infectious diseases (such as droplets and contact infections). Therefore, this study uses commuting data, instead of adjacency data, as geographic information (Figure 1). Adding commuting data to our model can better capture epidemic situation in a region, which is important for public health organizations.

To consider inter-regional flows of people, we used commuting data from 47 prefectures in Japan. The data were provided by the national census report [56] in 2015. The provided data include the single daily average numbers of commuters from one prefecture to another, throughout all the days of the weeks. For the experiment in each year, we represent the data as a graph G = (V, E, W) in the proposed model because the national census report provides only the number of commuters, regardless of the year. We divided the number of commuters by the maximum number of commuters between every prefecture pair, which is known as the min-max normalization to graph-edge information, such as W. The maximum number of commuters (270,000) travels from Kanagawa prefecture to Tokyo prefecture. Moreover, 135,000 commuters travel from Osaka to Nara. The edge weight, representing commuters traveling from Osaka to Nara, is shown as 0.5 (=135,000/270,000) in the graph, after applying min-max normalization.

4. The results of this study are shown in Fig.4~6 until October 2019. Can the author provide a simulation to the first half of this year?

Thank you for your comment. We have added the experimental results of the prediction in the 31st week in 2019 to the 30th week in 2020 to all related figures and tables. In addition, we have added the description and discussions regarding the prediction result in 31th week in 2019 to 30th week in 2020 to the “Results and Discussion” section.

5. The number of references in 2019 and 2020 is very small (only 1 out of 50), please add the reference for the past two years.

Thank you for your comment. We have added more references from 2019 and 2020, especially to the “Related work” section, as follows:

P3 Lines 67-105

Related work

Influenza prediction

Influenza prediction methods can be broadly classified into three categories: compartmental model-based, statistical and time series, and machine-learning.

Compartmental models include ``susceptible--infected--removed'' (SIR) [10] and incidence decay with exponential adjustment [11] models. They differ from statistical and machine-learning methods because they set suitable parameters for each compartment and focus on understanding disease dynamics. Statistical and time-series methods include the autoregression-integrated moving average [12] and generalized autoregressive moving average [13] methods. In particular, the autoregression with Google search method (ARGO

method) [14], which is based on linear regression using the input data of the Google search time series and historical influenza-like illness data, has exhibited superior results for flu forecasting [15-17]. Our GCN-based model is based on machine learning. Other examples of machine-learning methods include linear regression [18,19], random forest [20], Gaussian process [21], and long short-term memory (LSTM) [7,8,22].

Resource selection for the prediction method is also an important factor in influenza prediction. Many studies rely on user-generated content (UGC) from internet services, such as search services [12,14,23,24] and social networking services [25-27]. Infectious disease surveillance conducted with online content, as listed above, is generally described as infoveillance [28]. Currently, Google Flu Trends [24] is one of the most representative systems, which is designed to estimate the current influenza-like illness rate using related Google search terms. Signorini et al. [29] examined the volume of tweets that included keywords related to influenza in the Twitter stream, and showed the usefulness of Twitter data for tracking flu epidemics. Besides user-generated content, many studies used diverse resources to improve models: Wikipedia [30], historical flu data [14,31-33], and weather data [34].

Our model used historical flu data as a resource. Moreover, our research on influenza prediction for each prefecture is related to [5,6,35,36]. Senanayake et al.[5] used a kernel function based on the distance between two areas to capture spatial dependence. Wu et al. [6] used a convolutional neural network (CNN) architecture to convolve the information of surrounding areas. In contrast to the studies mentioned above, our study used regional commuting data to model the flow of people into a specific area. Brockmann et al. [35] attempted to capture the onset of an epidemic using data on international traffic. Wang et al. [36] extended the classic SIR model to consider the visitor transmission between any two areas to predict the intra-city epidemic propagation using the traffic volumes in the cities. To the best of our knowledge, our study is the first attempt to predict the influenza volume in detail for a large area (such as the entire territory of Japan) by considering the inter-regional flow of people using machine learning.

16. Lu FS et al. Improved state-level influenza nowcasting in the United States leveraging Internet-based data and network approaches. Nat Commun. 2019;10(1): 1–10.
17. Ning S et al. Accurate tracking of regional influenza epidemics using Internet search data. Sci Rep. 2019;9(1): 1–8.
18. Aiken EL et al. Real-time estimation of disease activity in emerging outbreaks using internet search information. PLoS Comput Biol. 2020;16(8): e1008117.
24. Zou B et al. Transfer learning for unsupervised influenza-like illness models from online search data. In Proceedings of the World Wide Web Conference, 2505–2516 (2019).
32. Wang L et al. DEFSI: Deep learning-based epidemic forecasting with synthetic information. In Proceedings of the AAAI Conference on Artificial Intelligence, 9607–9612 (2019).
33. Wu N et al. Deep transformer models for time series forecasting: The influenza prevalence case. arXiv:2001.08317 2020 [cited 21 Oct 2020]. Available from: https://arxiv.org/abs/2001.08317

To Reviewer #2:
Major concerns:

> 1. One key contribution of the study should be the consideration of 'periodicity in a time series' (page 2, line 51) in the model, which was neglected in the previous Zhu et al. [18] Encoder-Decoder model. But, the authors did not explain what is it, and why it is important. Since the study did not use time-dependent dynamic commuting data, I would 'guess' the periodicity is in the weekly disease data. The authors should not let readers to guess, thus they should explain and clarify the 'periodicity' term where they first mention it, and emphasize the consequence of neglecting it; and which would help emphasizing the contribution of this study.

Thank you for your comment. We agree that it is better to add a more detailed explanation of “periodicity in a time series” and why it is important.
We have added more descriptions to clarify the term “periodicity” to the “Introduction” section as follows:

P2 Lines 38-57
Introduction
Furthermore, we estimate the suitable uncertainty of our model's prediction using a prediction interval. This is important for decision-making in public health (such as vaccine demand or allocating medical personnel). Our spatiotemporal model is based on neural networks that are adopted by some epidemic prediction studies [6,8]. However, it is difficult to estimate the prediction interval for the downside or upside of prediction points because neural networks conduct point estimation. Therefore, owing to the unknown reliability of prediction results, certain decisions become difficult for public health authorities to make. To resolve such difficulties, Zhu et al. [9] presented an encoder--decoder method with an inference of prediction intervals by calculating three sources of prediction uncertainties, model uncertainty, inherent noise, and model misspecification, using Monte Carlo (MC) dropout, which was derived from the property of dropout-approximate Bayesian inference. This method appends an inference module on a trained model, without re-training it, to estimate the prediction uncertainties of the model. However, Zhu's method tends to favor a prediction interval that is much larger than adequate for non-epidemic periods (mainly in summer). This is due to the lack of consideration of one-year periodicity in time series; flu data holds strong seasonality (i.e., epidemic in winter and non-epidemic in summer). In brief, Zhu's method is not suitable for time series with periodicity, such as flu data. Therefore, we extend their method to estimate a suitable prediction interval for one-year cyclic trends in time series and evaluate the effectiveness of the extended method.

> 2. According to the dataset description, the commuting data is in 'the daily average number of commuters from one area to another area'. Is this dataset differentiate weekdays/weekends, or from Monday to Sunday? The time unit for the model is by weekly basis, how did the daily data converted to weekly before the 'min-max normalization'?

Thank you for your comment. The commuting data were taken from the survey results aggregated by the Japanese government, and these data include daily average numbers of commuters between any two prefectures. The data showed no distinction between weekdays and weekends. We divided the number of average daily commuters between a certain region pair by the maximum number, which is the ratio of the number of pairs to the maximum number. As an edge weight from a region to the other, this ratio works as the weight coefficient, indicating the strength of influence from one region to the other. Neural networks are intended to learn optimal parameters from training data, along with these edge weight coefficients, to compute how much the former region affects the number of patients in the latter region in the succeeding week.
We have added a more detailed description of commuting data in the "Datasets" subsection of the "Experiment" section as follows:

P10 Lines 277-295
Commuting data
Spatiotemporal models typically use adjacency and distance between observation points to model geographic information. However, human mobility is strongly linked with the transmission of infectious diseases (such as droplets and contact infections). Therefore, this study uses commuting data, instead of adjacency data, as geographic information (Figure 1). Adding commuting data to our model can better capture epidemic situation in a region, which is important for public health organizations.

To consider inter-regional flows of people, we used commuting data from 47 prefectures in Japan. The data were provided by the national census report [56] in 2015. The provided data include the single daily average numbers of commuters from one prefecture to another, throughout all the days of the weeks. For the experiment in each year, we represent the data as a graph G = (V, E, W) in the proposed model because the national census report provides only the number of commuters, regardless of the year. We divided the number of commuters by the maximum number of commuters between every prefecture pair, which is known as the min-max normalization to graph-edge information, such as W. The maximum number of commuters (270,000) travels from Kanagawa prefecture to Tokyo prefecture. Moreover, 135,000 commuters travel from Osaka to Nara. The edge weight, representing commuters traveling from Osaka to Nara, is shown as 0.5 (=135,000/270,000) in the graph, after applying min-max normalization

3. Also about the description of the commuting data (page 9, line 276-282), the authors describe the number of commuters as 'inflow of commuting data', e.g. the 270,000 and 135,000, as the number of commuters from one area to another. Based on the terminology from graph theory and social network analysis, the term 'inflow' could indicate the total number of people/commuters go to a target area, e.g. the total number of people go into Tokyo from any area; and the counterpart 'out-flow' could mean the total people leaving from the area. The usage of term 'inflow' is misleading.

Thank you for your comment. We have revised "inflow of commuting data" to "the number of commuters" in response to the comment, as shown in the previous answer.

4. Following the #3 point, the input data for the model should be a weighted directed matrix (as suggested in figure 1). Commuting data is expected to be the number of people commute from the home area to work area. The people eventually will go back to their home in daily basis, i.e. a reversed direction flow relationships, or transpose matrix of flow matrix. Why the reverse direction of commuting flow is not considered and processed in the model? And, why direction of flow matters in the machine-learning based model?

Thank you for your comment. As for your question: "Why the reverse direction of commuting flow is not considered and processed in the model?", our model already uses both flows on commuter graph edges. Specifically, Equation (2) represents how our model captures both flows: $(D_O^{-1}W)^k$ indicates the direction of commuting flow, and $(D_I^{-1}W^T)^k$ indicates its reverse-direction flow. In addition, we explained this in the sentences between Equations (2) and (3): "*Furthermore,* $(D_O^{-1}W)^k$ *and* $(D_I^{-1}W^T)^k$ *represent the transition matrices of the diffusion and reversed processes, respectively, when considering both flows of people*."

As for your question: "Why direction of flow matters in the machine-learning based model?", our commuter graph represents commute data from the Japanese government, and we designed it as a directed graph to fully express the information available in the report. As you pointed out, we are also aware of the importance of both people flows, i.e., go to work and return home, with different parameters learned in the training phase of our method because these two directions might affect the regions, possibly, with different strengths of impact. Thus, our learning method uses both directions by transposing weight matrix W with different learned parameters; the graph must be represented as directed. There are two directed edges between the two regions A and B, i.e., one is for the residents of A who commute from A to B, and the other is those of B who commute from B to A; the two edges represent different weights. This information cannot be represented by an undirected graph. To clarify this, we added such explanation in the "Diffusion graph convolutional network" subsection of the "Material and method" section as follows:

P6 Lines 165-171

Diffusion graph convolutional network

DGCN uses the diffusion process in Equation 1 to model the diffusion convolution operation over graph input $\boldsymbol{X}$ *and filter* f_θ*, to capture the flow leaving and entering each region.*

$$\boldsymbol{X}_{:,m} \star \mathcal{G} \boldsymbol{f_\theta} = \sum_{k=0}^{K-1} \left(\theta_{k,1} \left(\boldsymbol{D}_O^{-1}\mathbf{W}\right)^k + \theta_{k,2} \left(\boldsymbol{D}_I^{-1}\mathbf{W}^T\right)^k \right) \boldsymbol{X}_{:,m} \quad m \in \{1...M\} \tag{2}$$

Here, $\theta \in R^{K \times 2}$ *are the filter parameters, and* g *denotes a graph convolution operation. Furthermore,* $(D_O^{-1}W)^k$ *and* $(D_I^{-1}W^T)^k$ *represent the transition matrices of the diffusion and reversed processes, respectively, when considering both flows of people. Our machine learning method uses both directions; it learns different parameters for each transition matrix. These two directions might affect the epidemic situation in regions with different strength of impact; thus, the input graph must be directed.*

5. Both figures 5 and 6 suggested that all models' predictions were lower than the true values at the peak of trends, especially the second peak (near 2018 10th). Why they all failed to capture the peak values? Why LSTM's peaks were almost all earlier than the true value, whereas CNN-Res were always later?

Thank you for your comment. As for your question: "Why they all failed to capture the peak values?", we agree that almost all the models' predictions were lower than the true values at the peak of trends in the results of 2018. However, we expanded our experimental results with 2020 data, where the situation was reversed, as shown in the revised manuscript. The expanded data in Figure 5 clearly indicate that in the seasons when epidemics grew much larger than in the previous years (such as 2018), the prediction model underestimates the peak value; whereas the seasons when the epidemics remain on a smaller scale than in the previous years, it overestimates the peak value (as in the season of 2020). This tendency is more or less true for all prediction models that use past data, including ours, because they are designed to learn the data from the most recent years.
As for your next question: "Why LSTM's peaks were almost all earlier than the true value, whereas CNN-Res were always later?", we are aware that the results in Figures 5 and 6 show such a tendency. However, Figures 5 and 6 show the prediction results in only one prefecture, and we confirmed that such a tendency did not always emerge in other prefectures. What is most remarkable in Figures 5 and 6 is that our proposed model almost always approximates the curve of the true values. In particular, it correctly detects the week of sudden outbreaks, although it underestimates the peak value in 2018 and overestimates it in 2020. This is mainly due to the improvement made by our GCN model based on commuting data.
We added an explanation of the tendency of all models' prediction from 2018 to 2020, in "RQ2: Effectiveness of the spatiotemporal model" of "Results and discussions" as follows:

P15 Lines 432-445
Results and discussions
RQ2: Effectiveness of the spatiotemporal model
Figure 5 shows the time series for Okayama with a relative MAE improvement compared with LSTM in four years. According to these results, GCN+Seq2seq w/ PF can catch the beginning of epidemics in specific regions. This is because it uses GCN to learn the effects of influenza epidemics from other prefectures.

All the models' predictions were lower than the true values at the peak of trends in 2018. In contrast, the results of 2020 seemed to be inversed; all the models' predictions were higher than the true values at the peak of trends. We assume that this tendency is due to the characteristics of machine-learning methods that are designed to learn the data of most recent years. Evidently, in the seasons when epidemics grew much larger than in the previous years (as in 2018), these prediction models tended to underestimate the peak value. Furthermore, the seasons when the epidemics remained on a smaller scale than in the previous years, the models overestimated the peak value (as in the season of 2020).

6. In page 3 line 91, the authors claimed that 'Our study is the first to predict the influenza volume in detail on a large area...'. But in fact, the model considered only 47 areas, which is not a large number and is a low resolution for the whole country. Practically speaking, the 47 areas (assumably prefectures) might be enough for national level management, but they are too large for local disease control or health management, therefore not so useful for 'regional public health organizations' (page 15 line 459). Is this model applicable to smaller areas (higher resolution, e.g. municipal)? If so, what should be prepared and which part should be modified; if not, why?

Thank you for your comment. We agree that our experiments for 47 prefectures might be enough for national-level management; however, we need to perform more experiments in smaller areas to confirm our method to be “useful for regional public health organizations.” We have revised the sentences that contain the expression “regional public health organizations” in the “Conclusion” section, which was not suitable for our experimental settings, as follows: “*The proposed model with uncertainty estimation will contribute to infection control measures of public health organizations*.”

As for the question: “Is this model applicable to smaller areas (higher resolution, e.g., municipal)? If so, what should be prepared and which part should be modified; if not, why?”, we intended our model to be applicable to areas of smaller size than the prefectures without modifying the model. We assume that our model can be applied to any area wherever we have two inputs: commute information between regions and observed numbers of past patients in the region, for building the model. However, such data are not always available, as few smaller local organizations aggregate and publish the data. This is a limitation of the current study, and it might be a subject of our future work to address the prediction tasks with limited data.

7. Following previous point, is it possible to extend/apply the model to be used in early warning system?

Thank you for your comment. We assume that it is possible to apply our model in an early warning system. For example, one of the existing flu prediction models based on a machine-learning method (https://fludetector.cs.ucl.ac.uk/) adopts a simple strategy in which the decision of warning is made based on the prediction value of the system; the warning is triggered when the value exceeds a certain threshold determined by experts. Such a strategy can also be applied to our model.

8. From the view of spatial epidemiology, the disease spread from one place to another, through droplets or direct/indirect physical interactions (etc.) and through the flow of the infected people. The infectious process is described as SIR model, which has (at least) three conditions: susceptible, infected, and recovered. The infected person go through the SIR process, and thus a time-lag is expected in the process, i.e. from susceptible to infected, and from infected to recovered. How does this machine-

learning based model(s) handle the complicated SIR (or SEIR, SLIR, SIS, etc.) process and the time-lag effect?

Thank you for your comment. Compartmental models, such as SIR and SEIR, consider **susceptible**, **exposed**, **infectious**, and **recovered** as states of patients, describing the infectious process using a few parameters. Such models greatly differ from our machine-learning method. The purpose of compartmental models is to understand the infection process, whereas that of our model is to achieve accurate prediction of the future number of infected patients. Our model predicts the observed **infectious** cases of compartmental models in the future utilizing historical influenza data and commuting data as input. Thus, it does not consider the time-lag effect from **susceptible** to **infected** and **infected** to **recovered**. The consideration of these effects is beyond the scope of our model. Our model achieves the best prediction performance of the future observation of **infected** cases by making the problem setting simpler, considering only a single compartment of patients.
We have added a description of the difference between machine-learning methods (such as ours) and compartment models (such as SEIR) in the "Influenza prediction" subsection of the "Related work" section as follows:

P3 Lines 67-81
Related work
Influenza prediction
Influenza prediction methods can be broadly classified into three categories: compartmental model-based, statistical and time series, and machine-learning.
Compartmental models include ``susceptible--infected--removed'' (SIR) [10] and incidence decay with exponential adjustment [11] models. They differ from statistical and machine-learning methods because they set suitable parameters for each compartment and focus on understanding disease dynamics. Statistical and time-series methods include the autoregression-integrated moving average [12] and generalized autoregressive moving average [13] methods. In particular, the autoregression with Google search method (ARGO method) [14], which is based on linear regression using the input data of the Google search time series and historical influenza-like illness data, has exhibited superior results for flu forecasting [15-17]. Our GCN-based model is based on machine learning. Other examples of machine-learning methods include linear regression [18,19], random forest [20], Gaussian process [21], and long short-term memory (LSTM) [7,8,22].

Minor concerns:

1. Table 2 presented the average MAE and R-squared of 47 areas. While the average values shows that their model (GCN+S2s w/ PF) are in overall outperform other models, the average values may be misleading by outliers. Thus, showing the distribution of the 47 values were needed, e.g. with std or boxplots. I believe these results could be presented using a set of boxplots (3 MAE and 3 R-squared), with vertical axis showing the MAE or R-squared, horizontal-axis showing the 1-to-5-weeks, and six boxes (different colors) for each week showing the values for 47 areas for the six models. Line plot with error bars can also be used to show the average and plus-minus standard deviation if boxplot is not clear.

Thank you for your comment. We agree that the average values of experimental accuracies may be misleading, owing to the existence of outliers; whereas boxplots showing the distribution of the prediction scores in each prefecture are better visualizations to solve the problem. We show the evaluation results from one to five weeks by six models (our model and other models in comparison) for a four-year period (2017, 2018, 2019, and 2020) using two metrics (MAE and R-squared) in 240 boxplots, in the "Supporting information".

2. Following previous point, it should be possible to calculate the MAE and R-squared in aggregated (national) level, instead of average of 47, and the national level results shall also be useful for discussion.

Thank you for your comment. In accordance with this comment, we will be able to calculate the MAE and R-squared of each model in the aggregated level of 47 prefectures from the experimental results. However, none of the models has been optimized to predict the future influenza volume at the national level. Considering this, we are afraid that the comparison of each model at a national level might be misleading to the readers, and we have refrained from showing these results in this paper.

3. The comparative model (GCN+S2s w/ AD) considered only the adjacent relations between areas (polygon shapes of the 47 areas). In transportation and spatial analysis, the strength of interaction between cities (e.g. flows) can be estimated mainly using gravity model or radiation model. In simple words, the interaction strengths were higher between closer cities, and lower between farther cities, i.e. distance decay effect. What if adding another comparative model that calculate the inversed distance as the weight matrix?

Thank you for your valuable comment. We absolutely agree that the model with the graph of the inverse distance between regions you recommend is a good mode to compare with ours. Thus, we called the configured model as "GCN+S2s w/ DD" and added a detailed description of the model to the "Compared models" subsection in the "Experiments" section as follows:

P11 Lines 322-332
GCN+Seq2seq w/ DD
To validate the effectiveness of PF as spatial information, compared with other geographical relations between prefectures, we considered the distance between the prefecture regions. We assume that the inter-region distance is an important factor in estimating the strength of the interaction between regions, along with geographical adjacency. We prepared the data by measuring the straight-line distance between the locations of the government offices of each prefecture.
In our model, we substituted the graph weighted by the inverse distance between regions, after min-max normalized, for commuting data. The closest distance's edge weight is shown as 1; smaller values indicate longer distances. We call this model with inverse distance data (DD) between 47 prefectures as ``GCN+Seq2seq w/ DD."

4. Page 12 line 378, what is 'examples of learned'. What do the colors means in Figure 3. Tokyo and Aichi were in the 'min' values, which should means that the interactions from Nara to Tokyo/Aichi are low, thus not important and could be ignored?

Thank you for your comment. We apologize for the lack of explanation in Figure 3. The visualization of Figure 3 was created utilizing the learned localized filter derived from Equation (2). Simply speaking, it represents how much influence our model received from other prefectures, except for the target prefecture (as shown by a star), as calculated based on the learned parameters of the model. Red indicates that the prefecture is given larger weights, meaning that it contributes significantly to predicting the epidemics of the target prefecture. Meanwhile, blue prefectures are given smaller weights, which makes less contributions to the prediction. To improve visibility, most prefectures are represented in white, given that their weights are less than 5% of the maximum. We added an explanation to the caption of Figure 3 as follows:

Caption of Figure 3
Visualization of the weights of learned localized filters of Equation (2) of (a) GCN+Seq2seq w/ PF, (b) GCN+Seq2seq w/ DD, and (c) GCN+Seq2seq w/ AD against the prediction target node (Nara prefecture shown as starred). The colors represent the weights, i.e., the strength of influence of each prefecture on the prediction of the target prefecture. The red prefectures are given larger weights, i.e., they contribute significantly for predicting the epidemics of the target prefecture, while blue prefectures are given smaller weights. Note that most prefectures are represented in white for visibility, as their weights are less than 5% of the maximum.

6. Figures 3 and 4, what is the purpose of the small squares at the corner of each map (not the Hokkaido area)?

Thank you for your comment. The land in the small square at the corner of Figure 4 is Okinawa prefecture, which consists of islands located towards the South-East of the main four islands of Japan. We have added an explanation to the caption of Figure 4 as follows:

Caption of Figure 4
Prefecture maps that illustrate the improvements of prediction accuracy measured by MAE in each prefecture, where the improvement ratios of the GCN+Seq2seq w/ PF against the LSTM are represented by colors. Red denotes improved prefectures, and blue denotes degraded prefectures. Prefectures enclosed in red and blue frames denote the best and worst five prefectures in each year, respectively. The small square at the corner of each map shows Okinawa prefecture.

5. Figure 4, consider adding legends on maps. And since the authors presented a map for a year, it would be better to show the 'improvement' percentage in all 47 areas with color ramp, and maybe used colored thick borders to highlight the highest and lowest five areas.
7. Table 1, consider align the second column (Definitions or Descriptions) to left.

8. Figures 5 and 6 consider changed x-label to Weeks from Date.

Thank you for your comments. We have modified Table 1 and Figures 4—6 according to your comments.

9. Finally, while the content is quite rich, the English writing in the manuscript is not publishable; some of the sentences needs to read twice or more to understand/guess the authors meaning, e.g. the above point 4 (examples of learned?), page 13 line 416 (beginning of epidemics?). It would be difficult for readers to understand the idea/method/uniqueness/contribution of the study, and possibly lead to misunderstanding. Please revise the writing.

Thank you for your comment. We carefully looked through our manuscript to improve the presentation and asked a professional English proofreader to revise the manuscript. We hope that the manuscript is much easier to follow than the previous version.
