# Supplementary material for: Predicting regional influenza epidemics with uncertainty estimation using commuting data in Japan
Source: PLoS One. 2021 Apr 22;16(4):e0250417. doi: 10.1371/journal.pone.0250417 (PMC8062106; doi:10.1371/journal.pone.0250417)
Supplement: Attachment — Submitted filename: Plos_one_reply.pdf [file pone.0250417.s004.pdf]

March 6, 2021

Dr. Tzai-Hung Wen
Academic Editor
PLOS ONE

Dear Editor:

Thank you for inviting us to submit a revised draft of our manuscript (PONE-D-20-20268), entitled "Predicting Regional Influenza Epidemics with Uncertainty Estimation using Commuting Data in Japan" to the PLOS ONE. We appreciate the time and effort you and each reviewer have dedicated to providing insightful feedback to help strengthen our manuscript. Thus, it is with great pleasure that we resubmit our manuscript for further consideration. We have incorporated changes that reflect the detailed suggestions you have graciously provided. We also hope that the edits and the responses we have provided satisfactorily address all the issues and concerns you and the reviewers have noted. We have also sought the help of a native English editor to improve our manuscript in terms of language and grammar. In this rebuttal letter, the comments or suggestions from you and the reviewers are inside black textboxes, our responses follow these, and our revisions to the manuscript are shown in italics.

Best regards,
Taichi Murayama

To Reviewer #3

> 1. The graph element is very crucial in this study, and thus relevant information should be given as clear as possible. For example,…

Thank you for this assessment. We agree that the information related to graph elements and GCNs should be clearer for unfamiliar readers. We answer your questions as follows:

> 1-a. why is the diffusion graph (Pg5) needed while the graph information has already given (Pg 9, "Commuting Data").
> 1-b. Is the diffusion process is inherence process of GCN or else?

1-a

In this paper, a diffusion process is intended to model the spread of infection on a commuting graph, representing the geological dependency of the flow of people. As the influenza virus infections considered in this study are only mediated by human bodies, we assume that the volume of infection positively correlates with the flow of people.

We represent the regional network as a weighted directed graph G = (V, E, W), where W is a weighted matrix representation of "commuting data", i.e., commuting volume between regions. A commuter only goes to another region and back and repeats this every day; however, the virus infection spreads beyond these two regions, i.e., to the neighboring nodes in G. The diffusion process is a random walk on G, and its transition matrix is given as $D_O^{-1}W$. Intuitively, it is a stochastic process of the "flow of viruses" through regions step-by-step; one day, a commuter transmits a virus to a region, and over the following days, other commuters transmit the virus from this region to other regions with some probability, and so on.

We tried to improve the explanations so that the manuscript is easier to follow for readers with diverse backgrounds. For readers who might seek more thorough guidance in this domain, we also added the following two citations to the manuscript:

[1] Molitierno, JJ. Applications of combinatorial matrix theory to Laplacian matrices of graphs. CRC Press (2016).
[2] Klicpera, J, Weißenberger S, Günnemann S. Diffusion improves graph learning. In Proceedings of NeurIPS (2019).

1-b

In principle, not restricted to a graph convolutional network (GCN), any graph-based algorithms that compute the influence from non-neighboring nodes with multi-hop separation necessarily adopt a random walk, diffusion process, or some related stochastic process. "The diffusion process" is considered as a generic term that refers to such a stochastic process that is implemented as a message passing between nodes. A graph spectral approach is another way to formulate the same problem using eigenvalue decomposition. Matrix factorization approaches might be considered as another example of non-stochastic process approaches.

A GCN adopts a graph convolution operation that can be either spectral or spatial (namely, message passing) depending on the approach it adopts. We adopted the latter approach, i.e., diffusion graph convolution because despite its theoretical soundness, spectral graph convolution suffers from problems such as inefficient implementation, difficult scalability, and poor adaptability.

We have rewritten the related sentences in the "Diffusion graph convolutional network" subsection of the "Materials and Methods" section to make these points clearer:

Diffusion graph convolutional network

We used a diffusion GCN (DGCN), which was originally developed for traffic flow prediction by [51], where we modeled the spatial dependence of the virus spreading by applying a diffusion process, i.e., random walk on a commuting graph. Thus, the temporal dynamics of the infection spread through regions were captured by a stochastic process on the input graph G.
Intuitively, this stochastic process represents the step-by-step "flows of viruses" through regions; one day, a commuter transmits a virus to a region, and over the following days, other commuters transmit the virus from this region to other regions with some probability, and so on. The transition matrix of the diffusion process is $D_O^{-1}W$*, where* $D_O^{-1} = diag(W1)$ *is the diagonal matrix of the total out-commuters from each region, and 1 denotes the all-ones vector. The stationary distribution of the diffusion process is as follows:*

...Equation (1)

where k represents the number of diffusion steps and $\alpha \in [0, 1]$ *represents the restart probability, with which the diffusion process restarts from its initial states [52, 53].*
The DGCN adopts a graph diffusion convolution using the above-mentioned diffusion process in Equation 1 over an input epidemiology signal X and a filter f_θ*, leveraging the flows both leaving and entering each region. The signal information X, such as the current number of patients, is transferred from one node to its neighboring nodes with the probabilities given in the transition matrix, and the spread signal distribution can reach the above-mentioned stationary distribution after several steps. However, the DGCN uses only a finite K-step truncation of the whole diffusion process for computational efficiency. Thus, it captures the K localized graph structures of G as follows:*

... Equation (2)

[52] Molitierno, JJ. Applications of combinatorial matrix theory to Laplacian matrices of graphs. CRC Press (2016).
[53] Klicpera, J, Weißenberger S, Günnemann S. Diffusion improves graph learning. In Proceedings of NeurIPS (2019).

1-c. Furthermore, it seems that there are only single (cross-sectional) commuting data, since the articles states "…provides only the number of commuters, regardless of the year" (pg 9, "Commuting Data" section). Is that mean such information used throughout the GCN model, or as initial information and subsequently evolve through the diffusion process?

We used only single graph information, i.e., commuting data, to learn the proposed model. Through learning the model, the weights of the diffusion process (filter parameters θ in Equation (3)) are updated; however, the used graph information remains unchanged throughout both training and inference.

We have rewritten the related sentences in the "Task Definition" subsection of the "Materials and Methods" section to make these points clearer:

Task Definition
Additionally, we represent the regional network as a weighted directed graph G=(V,ε,W), where V is a set of nodes |V|=N, ε is a set of edges, and $W \in \mathbb{R}^{N \times N}$ *is a weighted matrix representation, such as the constant commuting volume between regions. The influenza prediction problem aims to learn the function f(·) that maps T' historical signals and a constant weighted matrix representation of G to T future signals:*

2. Recently, some study (see reference) also applied geographically weighted regression (GWR) into epidemic prediction. The reason that I raise this suggestion is that GWR also considers the spatial flow relation between regions which is similar in this study. This study may indicates GWR-based method may be improved using commuting data. Adding such information may be helpful for those researchers who using “statistical and time series” approach.

Thank you for your comment. We added a description of studies on the GWR model in the “Influenza prediction” subsection of the “Related Works” section as follows:

Related Works
Influenza Prediction
Moreover, our research on influenza prediction for each prefecture is related to the following studies. Senanayake et al. [5] used a kernel function based on the distance between two areas to capture spatial dependence. Wu et al. [6] used a convolutional neural network (CNN) architecture to convolve the information of surrounding areas. Liu et al. [37] used a geographically weighted regression model, which extended the ordinary linear regression model and embedded geographical location data into the regression parameters, with geographical information about hospitals, such as the number of hospitals per 10,000 population, to predict the COVID-19 situation in China. In contrast to the abovementioned studies, our study used regional commuting data to model the flow of people into a specific area. Brockmann et al. [35] attempted to capture the onset of an epidemic using data on international traffic. Wang et al. [36] extended the classic SIR model to consider the visitor transmission between any two areas to predict intra-city epidemic propagation using the traffic volumes in cities. To the best of our knowledge, our study is the first attempt to predict influenza volume in detail for a large area, i.e., the entire territory of Japan, by considering the inter-regional flow of people using machine learning.

[37] Liu F, Wang J, Liu J, Li Y, Liu D, Tong J, et al. Predicting and analyzing the COVID-19 epidemic in China: Based on SEIRD, LSTM and GWR models. PLOS ONE. 2020;15(8): e0238
